# Application of non-degradable waste as building material for low-cost housing

**DOI:** 10.1038/s41598-023-32981-y

**Published:** 2023-05-18

**Authors:** Siswanti Zuraida, Bart Dewancker, Romi Bramantyo Margono

**Affiliations:** 1 Graduate School of Environmental Engineering, Faculty of Environmental Engineering, The University of Kitakyushu, Kitakyushu, Japan; 2 Department of Architecture, Faculty of Environmental Engineering, The University of Kitakyushu, Kitakyushu, Japan

**Keywords:** Civil engineering, Sustainability

## Abstract

Building material is one of the essential aspects in accommodating the supply and demand of low-cost housing in Indonesia. Recently, several researchers have devoted much time and effort to developing waste recycling for building materials since it is more ecologically benign, particularly for non-degradable waste. This article focuses on recycling disposable diaper waste as composite material for a structural and architectural component of the building based on Indonesian building standards. In addition to offering a broad perspective on the implementation of experimental findings, the design scenario comprised the construction of low-cost housing with a floorplan area of 36 m^2^. The experimental results indicate that disposable diapers waste to use as composite materials of the building has a maximum capacity of 10% for structural components and 40% for nonstructural and architectural components. The prototype housing also reveals that 1.73 m^3^ of disposable diaper waste can be decreased and utilised for a housing area of 36 m^2^.

## Introduction

Low-cost housing is commonly understood as housing that is appropriate in quality and location. At the same time, it does not cost a level that prevents its occupants from meeting other essential living costs or affects people's fulfilment of fundamental human rights^1–4^. In most developing countries, access to appropriate and affordable housing is a present and growing issue. In some circumstances, the problem is not a shortage of housing but an inadequate source of income^1–3^. In other circumstances, income is relatively high, but home supply and financing are limited, making housing pricey^4,5^—nonetheless, the widespread implementation of self-help housing programs in developing countries limited applicability. While popular processes of self-construction and bottom-up development did occur, these did not provide a long-term or massive solution to the enormous housing demands.

The high costs of two crucial inputs of land and building materials are a fundamental reason housing needs to be more attainable for the urban poor. Building materials are often the single most considerable tangible input into the construction of housing and can account for up to 80% of the overall worth of a simple residential dwelling^6^. It leads to the cost factor becoming the first barrier to sustainable construction^7^. It is because building materials are essential to the structural integrity of the housing. To put it another way, if the price of building materials doubles in contrast to the median price of other commodities, the length of time that a household will be required to work to afford the price of building materials will also nearly double^8^. The latter is problematic since many governments, both central and municipal, continue to insist on using conventional building materials and techniques. The various building rules and regulations mandate these, the majority of which are either a holdover from the days of colonialism or were imported from other nations^9^. These restrictions and standards prevent using building materials that are more appropriate and readily available in the local area. Additionally, these prevent the use of construction technologies that are both cost-effective and environmentally friendly.

There is a need for policies that would broaden people's access to building materials that are both appropriate and economical. Similarly, it should financially support research and development efforts into cutting-edge building techniques. Construction plans and methods that are friendly to the environment and technology that are energy efficient and produce less pollution should be encouraged and made more readily available. In this regard, several researchers have examined various materials used for low-cost housing construction divided into natural fibres, earthen materials and industrial-building waste^10^. The most common building applications for natural fibre materials (e.g. rice husk, sisal fibre, and banana leaves) are panel board, reinforced composite materials, and insulation^10–13^. Therefore, the usage of lime and mud for nonstructural construction components like bricks for walls has become increasingly common when working with earthy materials^10,14–18^. Thus, making blocks out of raw mud has been developed further without including a burning step. Also, recycled materials by utilising waste from building materials^10,19–21^, such as steel and rubber and industrial materials^10,14,16,22^, such as fly ash become the best choice to lessen the impact on the environment and costs. Other ways of recycling non-biodegradable waste^15,23,24^, such as plastics, are considered low-cost materials. Furthermore, some researchers have developed user-friendly material that is accessible through a variety of inventions and technics, including compressed earth blocks^25–29^, dome construction^30–32^, rammed earth^33–35^, and vault construction^36–38^. Some even relate the advanced technologies^39^(e.g. intelligent construction site, simulation and modelling, digitalisation and virtualisation) to be used in construction sites and involving private organisation participation in infrastructure development^40^.

Like other developing countries, low-cost housing provision in Indonesia has been a serious concern in the last three decades as the urban population has grown at a pace of 4.1% per year, and it is predicted that 68% of Indonesians will live in urban areas by 2025^41^. The benefits of urbanisation are limited^41^ because of the problems that lead to more poverty, like the rise of slums because there are not enough affordable places to live^3,42^. The significant increase in the number of people living in cities has yet to consider how much land is available in cities, which has caused housing demand and land prices to go up. Due to the rapid urban population, Indonesia faces two significant consequences: housing demand and waste management.

In terms of housing demand, Indonesia has a large gap between supply and demand, with a demand for 780,000 units of housing per year and the capability of stakeholders to deliver 400,000–500,000 units per year^43–45^. A backlog of around 300,000 housing units every year must be resolved to provide homes for approximately 30% of urban residents who live in non-owned housing. Following government programs, housing provision is crucial, but building materials are limited. In Indonesia, concrete, bricks, wood, and ceramics continue to be the most used construction materials^46^ due to their large capacity and as mandated by the building rules and regulations. Nevertheless, regarding environmental considerations, those materials create new issues, such as clay bricks and tiles having the highest embodied energy^46^, carbon emissions, and eco-costs^47^.

Furthermore, population growth is accompanied by increased waste capacity in terms of waste management. According to statistics^48^, the total waste per year in 2019 was 29.21 million tons, which rose to 32.76 million tons in 2020. Due to the situation, the Indonesian government is focusing more on waste capacity management, decreasing roughly 17.68 million tons of waste in 2021. Population growth also causes an increase in the use of disposable diapers for baby care. Since its introduction in the 1960s, the popularity of disposable diapers has risen due to the benefits of the circular economy within various diaper versions that have been adapted for more comprehensive applications over time^49^. In addition, there is a social benefit, especially for parents, as performances are convenient and affordable.

For this reason, previous studies investigated using materials innovation from disposable diaper waste as composite materials. Unfortunately, recycling this waste is now restricted to scientific research. The general population does not understand it well, but according to studies, this material innovation has considerable benefits in structural strength, economy, and environment^50–55^. The research also demonstrated that the mechanical properties and microbial content of disposable diaper concrete, in specific compositions, are identical to conventional concrete^52,53,55^. Adding 1% diaper to concrete enhances internal curing hydration and produces the most robust, durable material^52^. In addition, a mixture of up to 5% disposable diapers with concrete had the maximum strength at 28 days compared to other percentages^53^.

Furthermore, sodium chloride can sanitise used disposable diapers from a health perspective^54^. Biological Oxygen Demand (BOD) and Chemical Oxygen Demand (COD) tests on concrete made from disposable diapers revealed minor differences with the inclusion of clean diapers^55^. Moreover, compared to other waste management methods such as incineration and co-firing, the recycling of disposable diapers as concrete components has more significant benefits regarding carbon emissions and eco-costs^51^.

As a result, the study intends to tackle the problem of housing provision by creating building materials from non-degradable waste, which is cost-effective while meeting building standards. This study was researched to get a fresh perspective on waste usage for building components considered low-cost housing components, and recycling disposable diaper waste is recommended. The research was then carried out through an experimental examination of composite materials of building components using disposable diapers as composite materials. The project is designed to capture the material use potential based on construction standards. The Indonesian Building Standard (*Standar Nasional Indonesia*/SNI) has become a standard regarding material mechanical and physical qualities.

## Methods

A laboratory experiment was undertaken to conduct a direct inquiry and To calculate the number of disposable diapers that can be used as construction components. The experimental study involved two kinds of composite materials, concrete composite for structural elements such as columns and beam thus mortar composite for architectural elements such as non-bearing walls and floors. The standard of building materials following Indonesian building standards and regulations (*Standar Nasional Indonesia*/SNI) as follows:

1. SNI 2847:2019 Structural Concrete Requirements for Buildings^56^
2. SNI 7656:2012 Mixed Design Procedures for Normal Concrete, Heavy Concrete, and Mass Concrete^57^
3. SNI 03-2834-2000 Technical Mixed Design for Normal Concrete^58^,
4. SNI 03-6882-2002 Mortar Specification for Building Materials^59^
5. SNI 03-0349-1989 concrete Bricks for Wall and^60^
6. SNI 03-0691-1996 Paving Block/Concrete Block^61^

### Identifying the calculation formula for composite materials

When changing or substituting composite materials in a concrete component, the difference in material densities must be considered. For instance, the method does not directly measure weight capacity by percentage when fine aggregate is substituted with disposable diapers. The apparent similarity between 300 g of fine aggregate and 300 g of disposable diapers is deceiving. Because disposable diapers are lighter than fine aggregates, 300 g occupy a much larger volume. It is necessary to convert the weight of materials using their densities to balance the capacity. The following formula is then used to obtain the maximum replacement capacity:

1 $${m}_{w}=\left(\frac{\begin{array}{c}{\uprho }_{w}\\ \end{array}}{{\uprho }_{fa}}\right)x {m}_{fa}$$

where${m}_{w}$ = mass of waste material capacity (g),${\uprho }_{w}$ = density of waste materials (g/cm^3^),${\uprho }_{fa}$ = density of fine aggregate (g/cm^3^),${m}_{fa}$ = mass of fine aggregate (g).

Consequently, the formula compromises as follows by adding the percentage of recyclable materials:

2 $${R}_{wc}={\%}_{rep}x\left(\frac{{\uprho }_{w} }{{\uprho }_{fa}}\right)x {m}_{fa}$$

where${R}_{wc}$ = capacity of recycled waste materials (g),${\uprho }_{w}$ = density of waste materials (g/cm^3^),${\uprho }_{fa}$ = density of fine aggregate (g/cm^3^),${m}_{fa}$ = mass of fine aggregate (g),${\%}_{rep}$ = replacement percentage of fine aggregate by waste material (%).

In this study, waste material is repurposed into disposable diapers, and the formula is created to:

3 $${m}_{d}=\left(\frac{{\uprho }_{d} }{{\uprho }_{fa}}\right)x {m}_{fa}$$

where${m}_{d}$ = mass of disposable waste diaper (g),${\uprho }_{d}$ = density of disposable waste diaper (g/cm^3^),${\uprho }_{fa}$ = density of fine aggregate (g/cm^3^),${m}_{fa}$ = mass of fine aggregate (g).

In consequence, the formula compromises as follows by including a percentage of recyclable disposable diapers:

4 $${R}_{dc}={\mathrm{\%}}_{rep}x\left(\frac{{\uprho }_{d}}{{\uprho }_{fa}}\right)x {m}_{fa}$$

where${R}_{dc}$ = capacity of a recycled disposable diaper (g),${\uprho }_{d}$ = density of disposable waste diaper (g/cm^3^),${\uprho }_{fa}$ = density of fine aggregate (g/cm^3^),${m}_{fa}$ = mass of fine aggregate (g).

### Determining standards for building materials

In order to be used as construction materials, the experiment's results must fulfil the appropriate building material criteria. The use and specifications for concrete based on compressive strength are displayed in Table 1. The standard distinguishes between three strengths of concrete, ranging from low to high. Low strength, with a minimum of 10 MPa, is often used for light building structural components. In contrast, high strength, with a minimum of 41 MPa, is employed for prestressed concrete and heavy building structural components. The experimental outcome will be categorised as the compressive strength of the samples.

**Table 1:** Strength and utilisation of concrete^62^.

Concrete type	Compressive strength (f_c_′) MPa	Strength code	Utilisation
High strength	≥ 41	K400–K800	Generally, it can be used for prestressed concrete such as piles, girders, concrete plats for runaway planes, and high-rise buildings
Medium strength	21–40	K250–< K400	Generally, it can be utilised for reinforcement Concrete, such as concrete plates for bridges, girders, precast curbs, culverts, abutments, and middle-rise buildings
Low strength	15–< 20	K175–< K250	Generally, it can be utilised for normal Concrete, such as cyclopean concrete, paving and housing, with a maximum two-story
	10–< 15	K125–< K175	Light structural components for building, landed housing, and base floor

The standard relates to concrete bricks and paving blocks whose strength is categorised into four categories for architectural components. The sample in this experiment followed a solid concrete brick between two types of concrete bricks: solid and hollow. Level I is the strongest, having a minimum strength of 10 MPa, and is commonly used for structural components such as bearing walls. The lowest level is level IV, which has a minimum strength of 2.5 MPa and is suitable for nonstructural components such as wall partitions. The highest level for paving blocks is A, which has a minimum strength of 40 MPa and is used for public roadways. The lowest level is D, which has a minimum strength of 10 MPa and is suitable for dwelling floors or garden pavement. Table 2 contains more information on architectural components and their applications.

**Table 2:** Strength Level and Utilisation of Concrete Bricks and Paving Blocks^60,61^.

Strength level	Average gross f_c_′		Gross fc' for each sample		Average gross f_c_′		Gross f_c_' for each sample		Utilisation
	kg/cm^2^	MPa	kg/cm^2^	MPa	kg/cm^2^	MPa	kg/cm^2^	MPa	
	Solid concrete bricks				Hollow concrete bricks				
Bricks									
I	100	10	90	9.0	70	7.0	65	6.5	Structural component fits for exposed structure
II	70	7.0	65	6.5	50	5.0	45	4.5	Structural component for a covered structure
III	40	4.0	35	3.5	35	3.5	30	3.0	Nonstructural components, covered condition and fit for non-plastered or exposed finishing
IV	25	2.5	21	2.1	20	2.0	17	1.7	Nonstructural components, covered condition and plastering
Paving blocks									
A	400	40	350	35					Public street
B	200	20	170	17					Pavement for vehicle parking
C	150	15	125	12.5					Pedestrian ways
D	100	10	85	8.5					Floor or pavement for garden

### Experimental procedure

**Figure 1:**
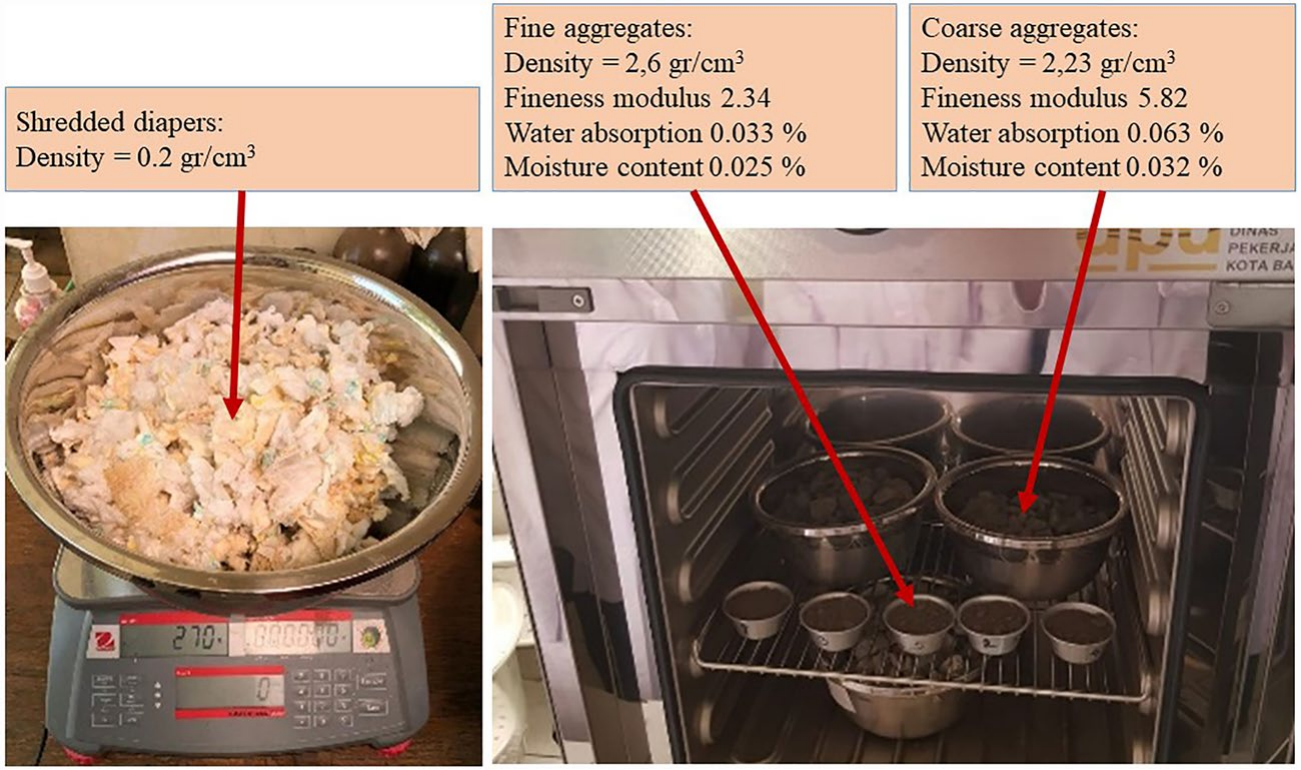
Physical properties of composite material.

During the experimental investigation, disposable diapers were substituted for fine aggregates to prepare composite material for construction components. The first step was to prepare used disposable diapers by washing, drying, and shredding them. Figure 1 depicts the aggregate test results, utilised as a base calculation of mix design for composite materials based on specific concrete and mortar mix design techniques. Following the aggregates test, Table 3 displays the mix of materials used to produce composite materials. It also discusses how the construction materials situation differed based on the structural and architectural components of the structure. Structural elements such as columns and beams pertain to the design of a concrete mixture, including Portland cement, fine and coarse aggregates, and water. In this experiment, the concrete mix design was initially formulated to achieve a maximum compressive strength of 25.00 MPa, which, according to Table 1, is typical for mid-rise structures. Refer to mortar mix design for architectural features such as walls and floors, where Portland cement, fine aggregates, and water are combined to form mortar.

**Table 3:** Samples composition of 1 m^3^ composite materials by recycling disposable diaper.

Mix design	Utilisation	Portland cement (kg)	Sand (kg)	Gravels (kg)	Disposable diaper (kg)
N_C_	Structural element: beam, column	370	747.9	892.6	0.00
C_5%_		370	745.02	892.6	2.88
C_10%_		370	742.15	892.6	5.75
C_15%_		370	739.27	892.6	8.63
C_30%_		370	730.64	892.6	17.26
N_M_	Architectural elements: wall, concrete bricks, floor	370	868.05	0.00	0.00
M_5%_		370	864.71	0.00	3.34
M_10%_		370	861.37	0.00	6.68
M_15%_		370	858.03	0.00	10.02
M_30%_		370	848.02	0.00	20.03
M_50%_		370	834.66	0.00	33.39

*N*_*C*_ normal concrete, *C*_*x%*_  concrete with x% replacement by disposable diaper, *N*_*M*_ normal mortar, *M*_*x%*_ mortar with x% replacement by disposable diaper.

The composite material samples were then subdivided based on their intended use in construction components, such as concrete samples meant for structural components such as columns and beams with sample of cubes (dimensions: 15 × 15 × 15 cm) and cylinders (height 30 cm, diameter 15 cm). While examples of mortar blocks measuring 5 × 5 × 5 cm were developed for architectural components such as walls and floors, they were shaped to resemble mortar blocks. After 28 days of curing, the compressive strength of a sample of six samples was evaluated.

### Determining low-cost housing standard

**Figure 2:**
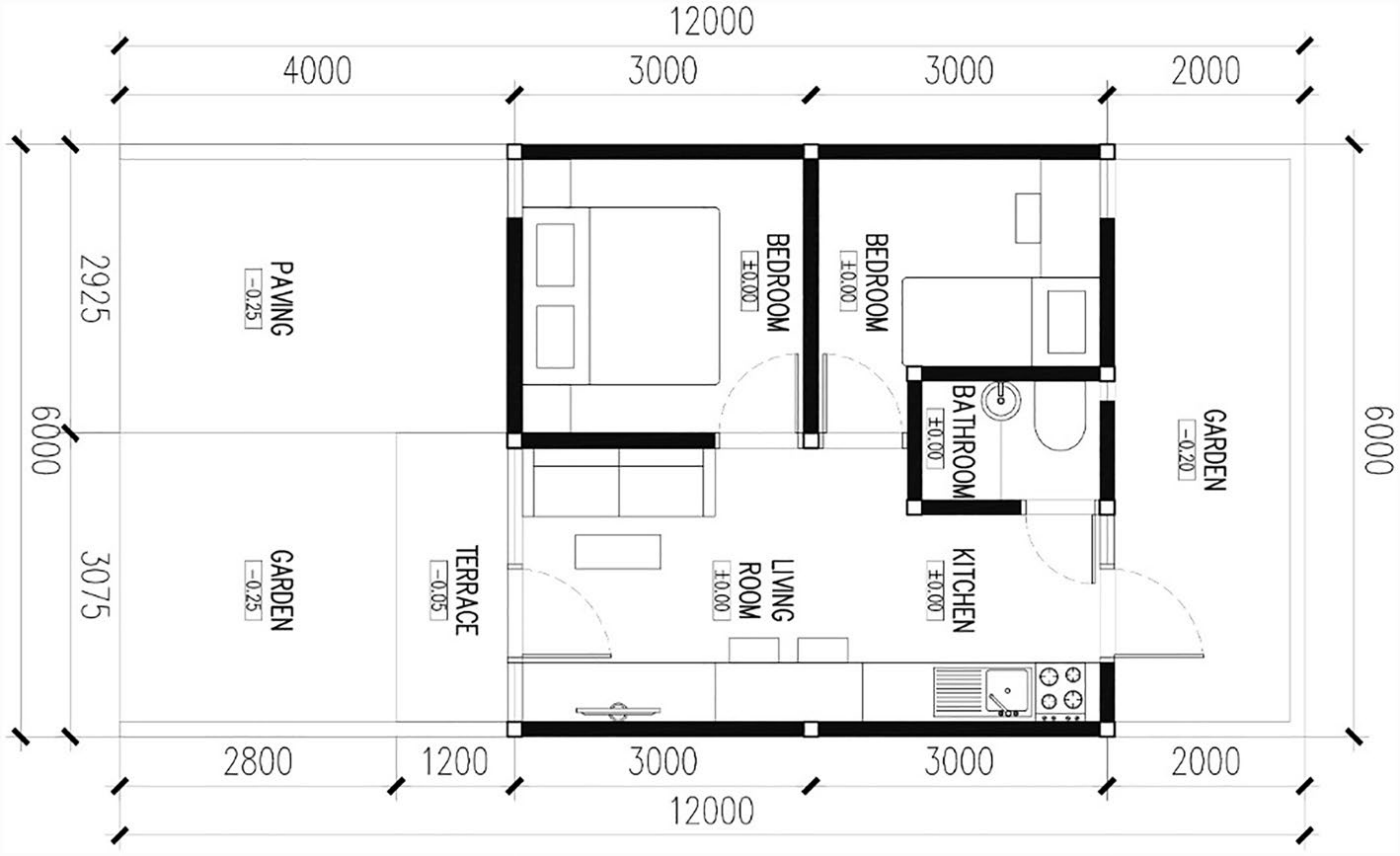
Floor plan of low-costs housing design in this study.

Since low-cost housing became a significant issue in this research, the housing standard for the implemented experimental study was designed by following standards of low-cost housing^63^. In this study, the housing is designed for four persons with a housing area of 36 m^2^ and a land area of 60 m^2^. The floor plan of the design is shown in Fig. 2 (Table 4).

**Table 4:** The standard for low costs housing in Indonesia^63^.

Standard per person (m^2^)	Area (m^2^) for three persons				Area (m^2^) for four persons			
	Housing area	Land			Housing area	Land		
		Minimum	Effective	Ideal		Minimum	Effective	Ideal
Minimum 7.2	21.6	60.0	72–90	200	28.8	60.0	72–90	200
(Indonesia) 9.0	27.0	60.0	72–90	200	36.0	60.0	72–90	200
(International) 12.0	36.0	60.0			48.0	60.0		

## Results and discussion

**Figure 3:**
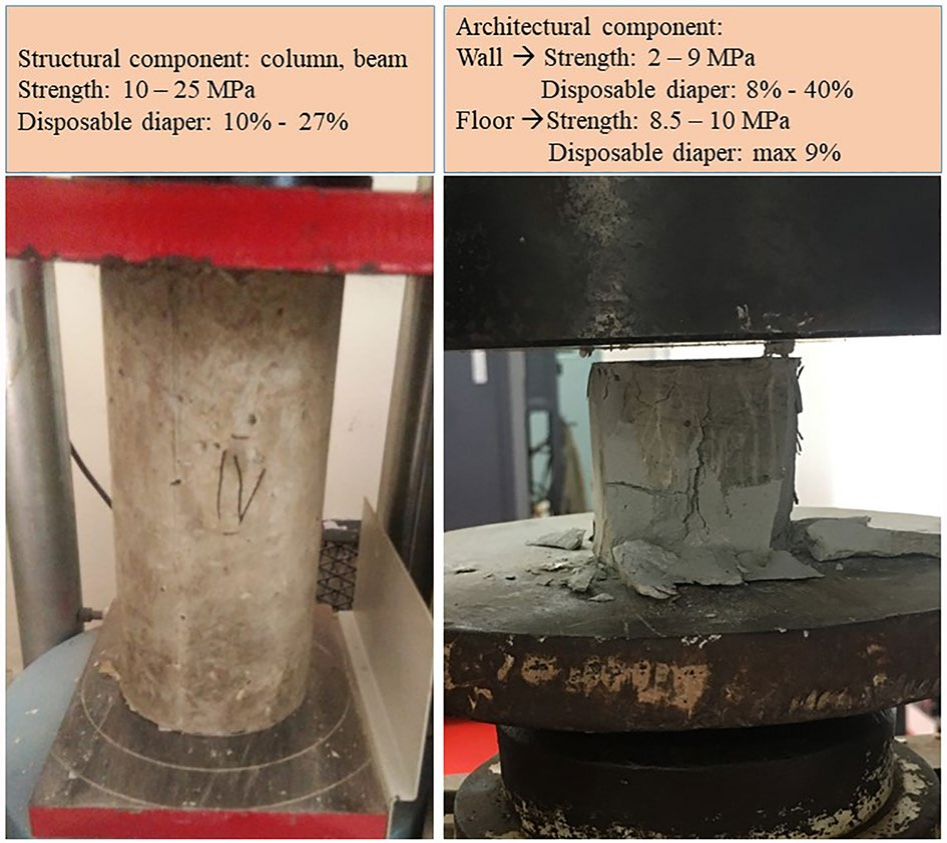
Tests on the strength of composite materials.

Figure 3 shows the test on the compressive strength of concrete and mortar specimens, and the findings are displayed in Table 5. Regular concrete attains a strength of 24.91 MPa, close to the mix design's intended strength of 25 MPa. However, the substitution of fine aggregates with disposable diapers resulted in a weakening of the structure as the number of disposable diapers increased. Similarly, a common phenomenon happened in the mortar mix design. The strength decreases as more disposable diapers are substituted for fine aggregates.

**Table 5:** Results for the compressive strength of samples of concrete and mortar.

Concrete type	Strength (MPa)	Mortar type	Strength (MPa)
N_C_	24.91	N_M_	11.36
C_5%_	23.07	M_5%_	8.05
C_10%_	22.48	M_10%_	6.79
C_15%_	17.39	M_15%_	6.03
C_30%_	7.9	M_30%_	5.25
-	-	M_50%_	1.11

**Figure 4:**
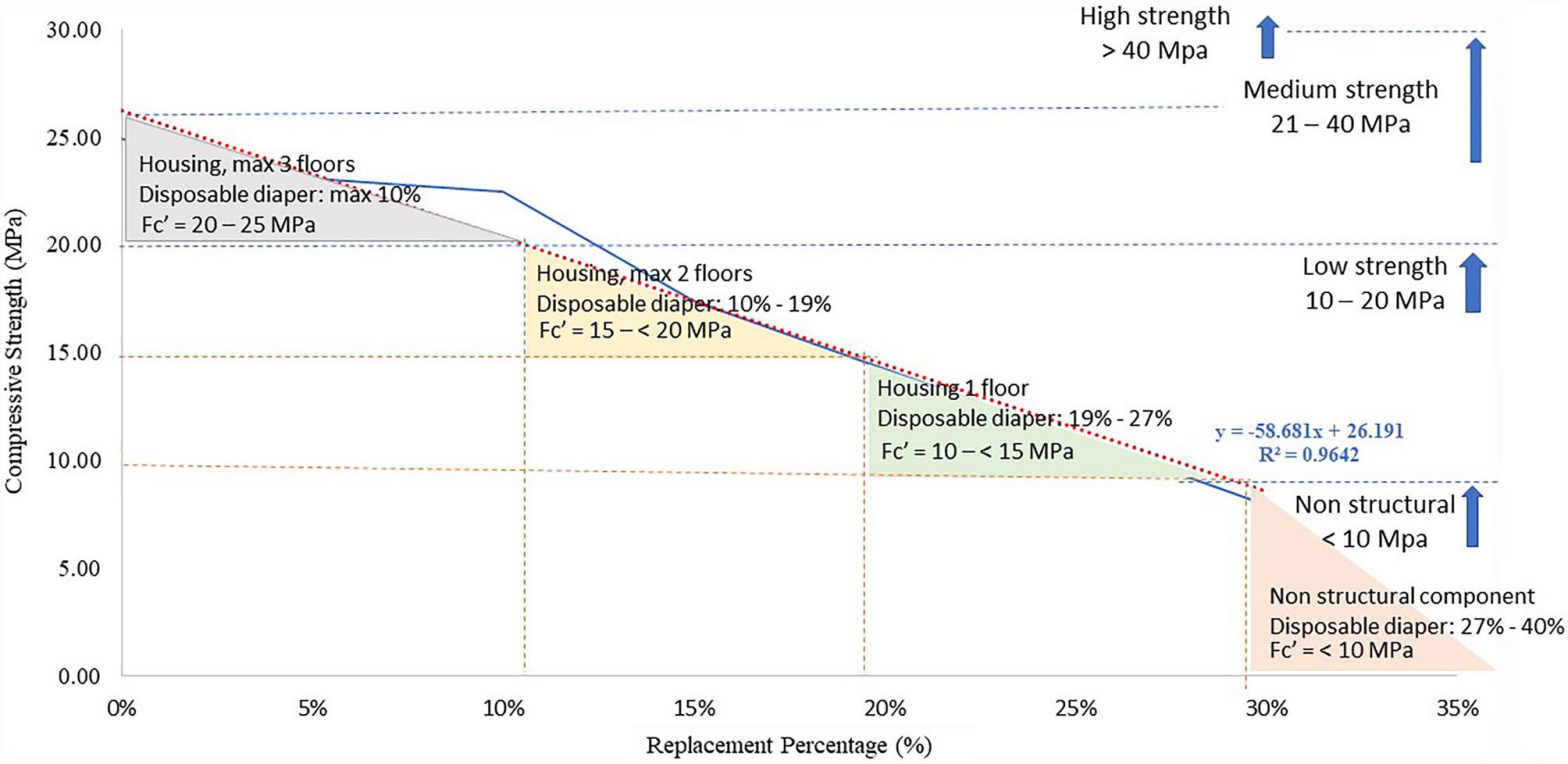
The utilisation of concrete with disposable diapers for housing component.

Consequently, the strength value is plotted to the linear regression equations by estimating the effect of disposable diaper replacement on compressive strength and considering their use as construction materials. As seen in Fig. 4, the employment of disposable diapers in concrete as a structural component is restricted by strength. The equation shown by linear regression is:

5 $$y = -56.681x + 26.191$$

where *y* is the compressive strength, and *x* is the percentage substitution of fine aggregate by disposable diapers. Concrete's strength can be predicted using the equation. In addition, by referencing Table 1 of the relationship between the strength and use of concrete, Fig. 4 illustrates the use of disposable diaper concrete for housing construction.

As shown in Fig. 4, the use is restricted to structural housing with a maximum of three-story, and a total replacement is 10%. The replacement range between 0 and 10% can achieve a strength between 20 and 25 MPa. However, for structural components, the maximum replacement rate is restricted to 27%, with a maximum strength value is 10 MPa. The maximum replacement rate for nonstructural components is likewise advised to be at most 40%. More than this proportion, concrete cannot be utilised for construction materials.

**Figure 5:**
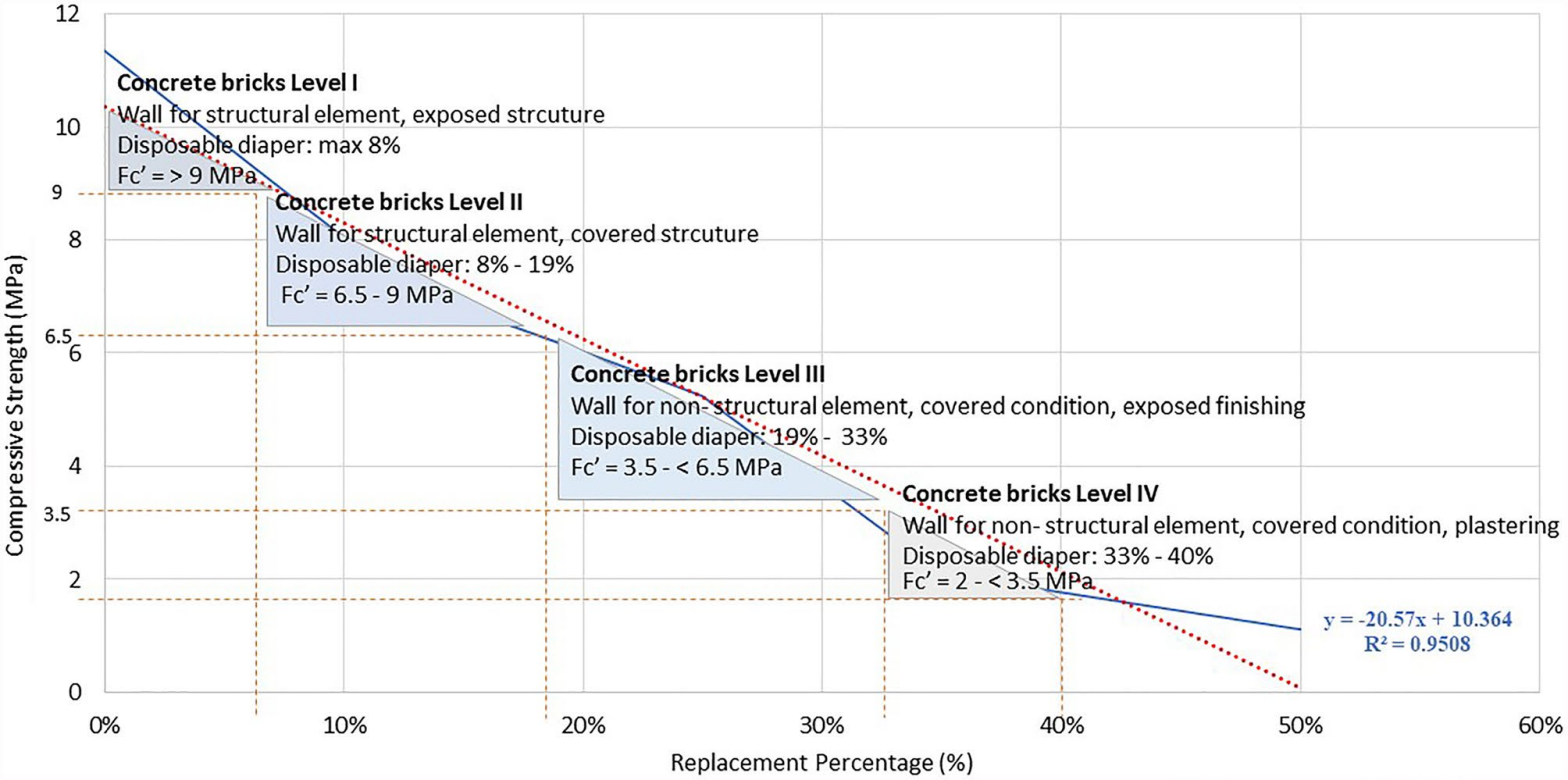
The utilisation of mortar using disposable diapers for concrete bricks.

**Figure 6:**
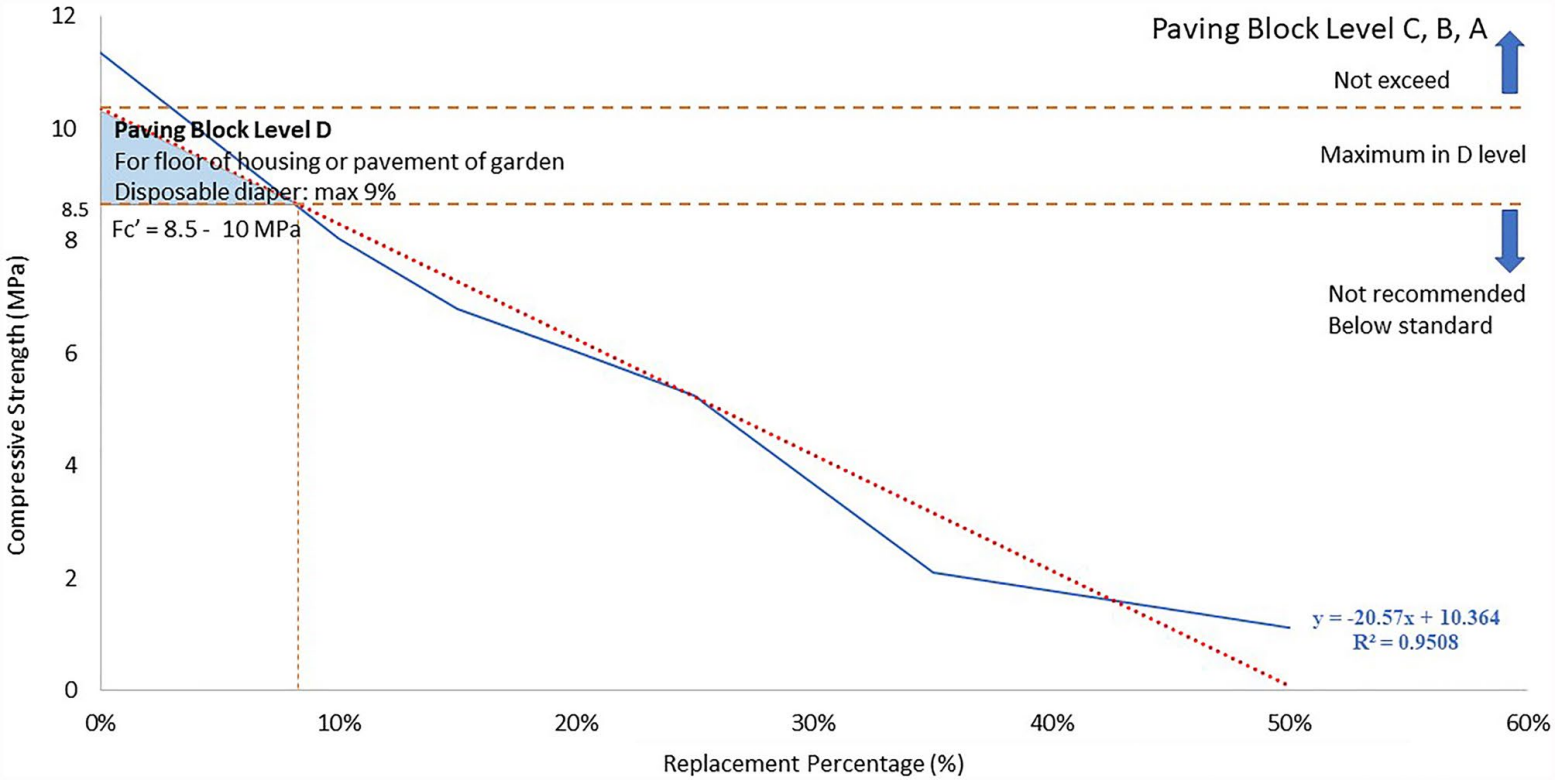
The utilisation of mortar using disposable diapers for road pavement or paving blocks.

The relationship between strength, percentage of replacement, and the use of mortar for architectural features is depicted in Figs. 5 and 6. In this study, the use is separated between concrete bricks and paver blocks based on Table 2 of strength standard and application as a construction material. In addition, the linear regression provided the equation for predicting the strength and percentage replacement of fine aggregates in mortar compounds:

6 $$y\hspace{0.17em}=\hspace{0.17em}-20.57x\hspace{0.17em}+\hspace{0.17em}10.364$$

where *y* is the compressive strength, and *x* is the percentage substitution of fine aggregate by disposable diapers.

As seen in Fig. 5, the strength of concrete bricks is ranked from I to IV, with I being the strongest and IV being the weakest. The maximum amount of disposable diapers that can be substituted for fine aggregates to achieve the level I strength is 8%, and for total replacement is 40% to achieve level IV as the lowest standard of concrete bricks. The replacement rate is restricted to 40%, and additional replacement is not advised because it is out of SNI 03-0349-1989^60^. Then, as is seen in Fig. 5, the maximum replacement of fine aggregate by a disposable diaper for a structural element such as a bearing wall under an exposed situation is 8%, whereas, under a covered state, the maximum replacement is 19%. In addition, more than 19% replacement can only be applied for nonstructural parts, with a maximum of 33% replacement for covered situations and exposed finishes. In addition, with a maximum of 40% replacement for plastered covered circumstances. Over 40% of disposable diapers do not meet specifications and are unsuitable for use in concrete bricks.

Then, for paving blocks depicted in Fig. 6, the material is restricted to only the D level, with a maximum replacement rate is 9%. It found that more than 9% substitution of fine aggregates with disposable diapers is not allowed for paving blocks due to the strength being below SNI 03-0691-1996 specifications^61^. Additionally, 9% of replacement is restricted to paving the floors of homes and gardens.

**Figure 7:**
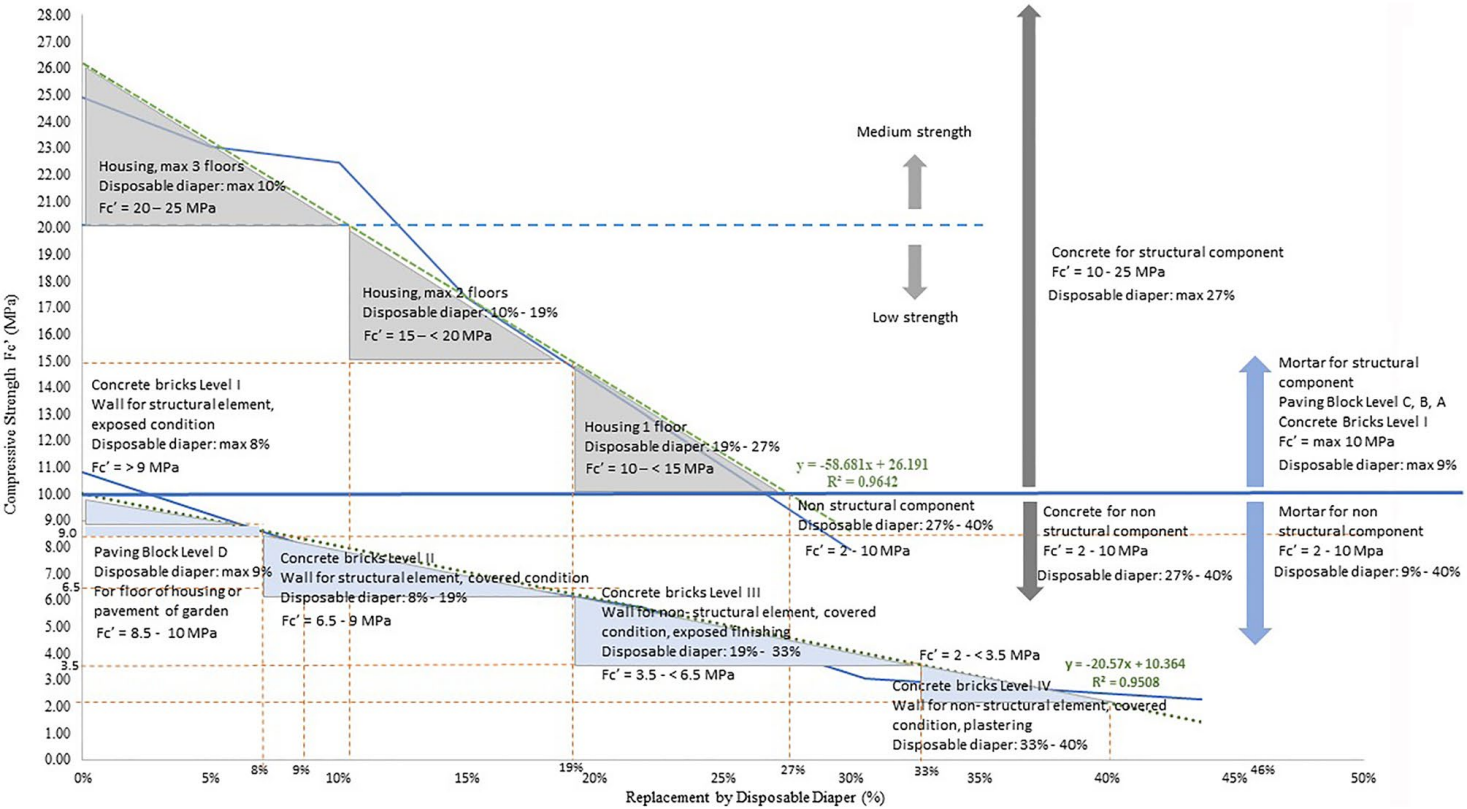
Summary utilisation of disposable diapers in composite materials for buildings.

Finally, to figure out comprehensive findings, the usage of disposable diapers on composite materials for the building materials is depicted in Fig. 7, which shows the various application of materials depending on their strength and component. In general, materials for concrete as structural components, such as columns and beams, can be substituted with disposable diapers to a maximum extent of 27% and a maximum strength of 10 MPa. For mortar as a structural component, such as bearing walls and public road pavement, the maximum replacement by a disposable diaper is between 8 and 9% with a strength of 8.5 MPa. Alternately, for maximal replacement, it can be used for nonstructural components with a maximum of 40% replacement and strength of 2 MPa. This application is for non-load-bearing wall partitions and low-impact floor pavers.

**Figure 8:**
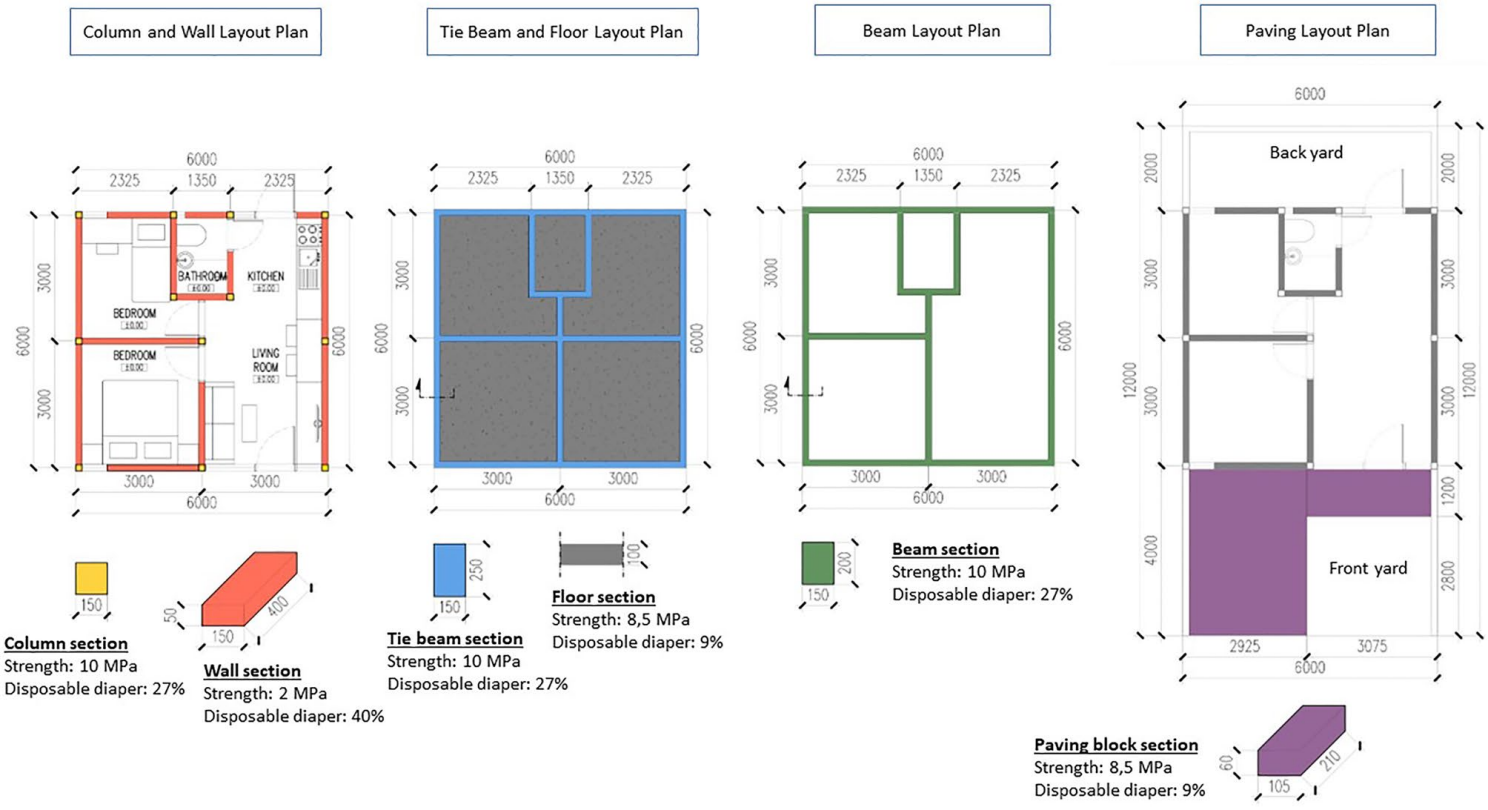
Layout plan for low-cost building materials utilising disposable diapers.

In addition, the result of the experimental study is applied to design requirements for low-cost housing based on Table 4 and Fig. 2. The use of composite materials in the structural and architectural components of a 36 m^2^ housing design is depicted in Fig. 8, with a maximum percentage of disposable diapers for housing components. For example, the maximum percentage of disposable diapers for column and beam structural components is 27%, with a strength of 10 MPa. The maximum percentage of disposable diapers for walls and floors is 40% and 9%, respectively, with a strength of 2 MPa and 8.5 MPa.

However, the structural analysis was not calculated exhaustively for this design scenario's column and beam size—the measurement related solely to the standard dimension of structural components for one-story housing in Indonesia. Further research and implementation of these findings must focus on more extensive structural assessments, including soil-bearing capability, load capacity, and other technical tests for structural analysis. Ultimately, to quantify the amount of composite material for the housing design, the volume of each construction component is determined using the floor design, and also accessible is the number of disposable diapers. The outcome is displayed in Table 6.

**Table 6:** The capacity of building materials to construct a type 36 low-cost housing prototype (36 m^2^).

Components	Type material	Strength (MPa)	Replacement by a disposable diaper (%)	Composite material (m^3^)	Disposable diaper (m^3^)
Column	Concrete	10.35	27	0.81	0.02
Beam	Concrete	10.35	27	1.09	0.03
Tie beam	Concrete	10.35	27	1.44	0.04
Wall	Mortar	2.14	40	16.36	1.57
Floor	Mortar	8.51	9	2.16	0.05
Paving	Mortar	8.51	9	0.94	0.02
	Total			22.79	1.73

According to Table 6, the total amount of construction materials required to build housing type 36 is 22.79 m^3^ with 1.73 m^3^ of disposable diapers. It indicates that a maximum of 7.6% of disposable diapers can substitute fine aggregate in construction. This finding gives insight into the effectiveness of the materials to be applied as building components in architectural design and further research. Also, by considering the environmental value of waste recycling, the material gives benefit to be developed on a large scale and by involving society and other stakeholders in collecting and managing the waste of disposable diapers.

### Study implication

Currently, the essential step in the recycling process for used diapers is to separate the plastic components from the organic fibres. It necessitates the execution of many complicated procedures, including collecting, crushing, sanitising, and sorting the components. Due to the difficulty involved in the process, only a few businesses are currently interested in recycling used diapers, such as Knowaste Ltd. United Kingdom^64^, Fater Ltd. Italy^65,66^, Diaper Recycling Technology Pte Ltd. Singapore^67^, Super Faiths Inc Unicharm Ltd. Japan^68^, and PHS Group United Kingdom^69^. However, the existence of the companies reveals that diaper recycling technology is currently only available in developed countries. It is primarily the result of two factors: differences in levels of expertise and access to equipment between developed and developing countries and a need for more awareness in developing countries regarding the potentially harmful effects of diaper waste^70^.

It becomes easily applicable by combining the waste as a part of composite materials such as concrete or mortar. Concrete is a widely used construction material due to its ease of processing, relatively low costs, and lack of high-tech manufacturing requirements. This research has concluded that adding used diapers to concrete does not significantly diminish its strength. It demonstrates that using used diapers to create composite materials is feasible, particularly concerning the development of environmentally friendly and cost-effective materials. Further, concerning this paper's social and economic advantages, the development of materials can be accessed from low to high technology. The procedures are relatively easy to conduct and low-cost. It also gives a comprehensive perspective of utilising disposable diaper waste as something valuable since it has ended up in the incineration process.

However, there are several limitations to implementing the findings broadly. To address the materials in wider applicable and in massive utilising, it needs the involving of stakeholders for waste treatment such as collecting the waste from households and washing the diaper waste until sanitising. The need for machines to shred the waste is also crucial to produce on a large scale due to low technology only being able to approach small-scale materials production. In addition, due to the existing various building rules and regulations only limited to conventional building materials, the role of government in regulating such materials needs to be opened.

At the same time, the limitations also give other benefits for future studies. The involvement of stakeholders and waste treatment mechanisms need to explore more to fill the gap. The innovation of shred machines for such materials can be challenging to be solved and invent. Moreover, to be implemented as low-cost housing, the materials need to be evaluated in terms of technical construction, cost, and housing price. This evaluation is proof to propose the materials in the financial mechanism of housing.

## Conclusion

The conclusion is drawn that using disposable diapers on composite building materials is represented by the linear regression equation *y* = − 56.681x + 26.191 for concrete and *y* = − 20.57x + 10.364 for concrete mortar. Where *y* is referred to compressive strength, and *x* is referred to the percentage replacement of fine aggregate by the disposable diaper. The utilisation is then divided into building components such as concrete utilisation covers column and beam with the maximum disposable diaper is 10% can obtain the strength of 20 MPa. This strength is appropriate for a three-story house. In contrast, a maximum utilisation of 27% is recommended for a single floor of housing with a strength of 10 MPa. The greater the replacement, the lower the SNI structural standard, and only recommended for nonstructural components.

In addition, in applying the composite mortar to wall and floor elements comprised of concrete bricks and paver blocks, respectively. The full replacement for a wall is 40%, resulting in 2 MPa of strength and classification as level IV concrete bricks, according to SNI 03-0349-1989. A full replacement of 9% is required for floor paving blocks, resulting in a strength of 8.5 MPa and compliance with level D of the SNI 03-0691-1996 standard for paving blocks. This maximum quantity is suitable for partition walls with no load-bearing capacity and floor pavers with low impact capacity. Replacement of more than this maximum capacity is not recommended for the utilisation of building materials. Incorporating experimental study findings into the design of low-cost housing, the total waste capacity of disposable diapers that can be utilised for single-story housing type 36 (36 m^2^) is 1.73 m^3^ out of a total composite material volume of 22.79 m^3^.

## Data Availability

The datasets generated during and analysed during the current study are not publicly available due to the patent process (Pat. Pend. No. P00202213376) but are available from the corresponding author upon reasonable request.
